# Draft Genome Sequences of 27 Northern Maine Clinical Isolates

**DOI:** 10.1128/MRA.01379-20

**Published:** 2021-02-04

**Authors:** Larry Feinstein, Kendra Batchelder, Lydia Tilley, Grace Stafford

**Affiliations:** a University of Maine at Presque Isle, Presque Isle, Maine, USA; b Mount Desert Island Biological Laboratory, Murawala Lab, Mount Desert Island, Maine, USA; c The Jackson Laboratory, Computational Sciences, Mount Desert Island, Maine, USA; Indiana University, Bloomington

## Abstract

We report the draft genome sequences of 27 common pathogens collected from a northern Maine hospital in 2017. These were sequenced in order to determine temporal and biogeographical patterns of antibiotic gene distribution. A total of 908 antibiotic resistance genes, 848 insertion sequence elements, and 57 plasmids were identified.

## ANNOUNCEMENT

Antibiotic resistance genes (ARGs) contribute to a significant public health issue, with ∼2.8 million antibiotic-resistant infections responsible for over 35,000 deaths annually in the United States (1). A total of 27 clinical isolates from the most common pathogens collected from the Aroostook Medical Center in Presque Isle, Maine, during winter 2017 were sequenced in order to determine the extent of ARG distribution and the mobile genetic elements that transfer them between bacteria. Institutional review board approval or ethics committee review was not required for this study. The isolate genera consisted of *Klebsiella* (8 isolates), *Escherichia* (6 isolates), *Pseudomonas* (4 isolates), *Enterobacter* (4 isolates), *Staphylococcus* (4 isolates), and *Stenotrophomonas* (1 isolate, originally identified as *Pseudomonas*). Table 1 provides additional information about each isolate sequenced.

**TABLE 1:** Data about each isolate sequenced

Species	Strain	Infection location	Genome size (bp)	GC content (%)	No. of reads	No. of contigs	*N*_50_ (bp)	Assembly accession no.	Raw read accession no.
Escherichia coli	EcPF15	Urinary tract	4,928,364	50.52	12,575,734	124	119,476	JACJHQ000000000	SRX9264872
E. coli	SE6-1	Urinary tract	5,052,494	50.75	12,901,582	106	142,608	JACJHP000000000	SRX9264873
E. coli	HB37	Urinary tract	5,124,920	50.52	11,305,112	90	221,887	JACJHO000000000	SRX9264883
E. coli	EcPF5	Urinary tract	5,131,891	50.41	11,746,384	122	334,530	JACJHN000000000	SRX9264892
E. coli	144	Urinary tract	4,962,945	50.62	11,293,956	127	189,818	JACJHM000000000	SRX9264893
E. coli	WP3-S18-ESBL-09	Urinary tract	5,057,591	50.62	11,986,542	119	153,491	JACJHL000000000	SRX9264894
Enterobacter hormaechei	RHBSTW-00564	Urinary tract	4,813,154	55.48	12,252,196	84	246,389	JACJHK000000000	SRX9264895
*E. hormaechei*	BW	Urinary tract	4,663,914	55.45	7,378,720	27	508,613	JACJHI000000000	SRX9264897
*E. hormaechei*	RHBSTW-00564	Respiratory	4,765,656	55.43	11,114,030	81	182,399	JACJHH000000000	SRX9264898
Enterobacter ludwigii	L140	Urinary tract	5,039,619	54.33	10,019,726	22	546,290	JACJHJ000000000	SRX9264896
Klebsiella aerogenes	HNHF1	Urinary tract	5,328,186	54.95	11,193,060	35	325,541	JACJHG000000000	SRX9264874
K. aerogenes	HNHF1	Urinary tract	5,287,865	54.97	10,112,974	45	320,838	JACJHF000000000	SRX9264875
Klebsiella pneumoniae	90-17	Respiratory	5,405,495	57.27	11,438,618	59	361,870	JACJHE000000000	SRX9264876
K. pneumoniae	MS14393	Urinary tract	5,286,397	57.48	9,976,828	46	589,446	JACJHD000000000	SRX9264877
K. pneumoniae	E16KP0288	Urinary tract	5,584,709	57.23	12,749,122	113	174,417	JACJHC000000000	SRX9264878
K. pneumoniae	SMKP03	Urinary tract	5,595,242	57.17	11,964,910	93	237,479	JACJHB000000000	SRX9264879
K. pneumoniae	Kp8701	Urinary tract	5,165,081	57.56	12,430,324	45	357,399	JACJHA000000000	SRX9264880
Klebsiella variicola	LEMB11	Urinary tract	5,481,031	57.44	12,292,892	28	576,709	JACJGZ000000000	SRX9264881
Pseudomonas aeruginosa	PA0750	Urinary tract	6,775,059	66.19	10,683,770	62	374,669	JACJGY000000000	SRX9264882
P. aeruginosa	PA0750	Respiratory	6,392,362	66.43	12,503,800	54	373,308	JACJGW000000000	SRX9264884
P. aeruginosa	PA0750	Wound lesion	6,601,920	66.17	11,790,784	108	376,348	JACJGV000000000	SRX9264885
P. aeruginosa	PA0750	Respiratory	6,832,764	66.06	1,923,108	77	258,204	JACJGU000000000	SRX9264886
Staphylococcus aureus	14505	Urinary tract	2,741,077	32.75	10,826,960	30	243,925	JACJGT000000000	SRX9264887
S. aureus	14505	Urinary tract	2,767,079	32.67	12,991,802	55	135,119	JACJGS000000000	SRX9264888
S. aureus	14505	Skin lesion	2,826,084	32.60	11,951,566	27	345,651	JACJGR000000000	SRX9264889
S. aureus	MJ015	Blood	2,736,988	32.74	13,200,774	32	290,137	JACJGQ000000000	SRX9264890
Stenotrophomonas maltophilia	NCTC10498	Urinary tract	4,577,239	66.42	13,956,7 24	44	202,957	JACJGP000000000	SRX9264891

Microbes were cultured in tryptic soy medium, and all incubations were 18 h at 37°C. We received strains on slants, and cultures were transferred to broth that was used to create streak plates. Pure cultures were obtained from isolated colonies on streak plates. Genomic DNA was extracted with an UltraClean kit (MoBio, Carlsbad, CA) and quantified with NanoDrop spectrophotometry (Fisher Scientific, Waltham, MA). Isolate cultures were stored in 25% glycerol at −80°C. The Jackson Laboratory (Bar Harbor, ME) constructed whole-genome libraries using the KAPA HyperPrep kit (Kapa Biosystems, Wilmington, MA), which sheared DNA into 150- to 350-bp fragments. Libraries were pooled and sequenced on a NextSeq sequencer (Illumina, San Diego, CA). Sequences were 2 × 150 bp with 100× mean coverage per sample.

All software utilized default parameters unless otherwise specified. FastQC 0.11.3 (2) was used for sequence quality control, and sequences were trimmed with Trimmomatic 0.33 (3). The Burrows-Wheeler Aligner MEM (BWA-MEM) algorithm (4) was used to align the sequences to reference genomes obtained from the RefSeq NCBI database (5). Contigs were assembled with SPAdes 3.10.0 (6), and NCBI’s Prokaryotic Genome Annotation Pipeline (7) was used for annotation. Standard features were called using GLIMMER v3.02 (8, 9) and Prodigal (10), and a scan was conducted for the following additional feature types: rRNA, tRNA, selenoproteins, pyrrolysoproteins, repeat regions, and CRISPR. The average (99.87%) and the lowest (99.65%) completeness scores were determined with CheckM (11). We used Spine (12) to identify core genomes for each species. Each isolate genome was compared to its core genome with AGEnt (12) to identify a core and accessory genome for individual isolates.

Accessory and core genomes were uploaded to the Comprehensive Antibiotic Resistance Database (CARD) (13) to identify any ARGs present in the sequences. A total of 908 ARGs were identified. The interleaved raw read files were analyzed using the plasmidSPAdes pipeline in SPAdes 3.12.0 (14) to identify plasmids. The contigs given by plasmidSPAdes were uploaded to CARD to identify ARGs. A total of 57 plasmids were identified, with 10 having at least one ARG. The assembled contigs were given to ISEScan (15) to identify insertion sequence elements. The nucleotide open reading frames were annotated using blastn (16). Gene functions were obtained with Uniprot (17). These data are being utilized to quantify temporal and biogeographical ARG distribution patterns in the northeastern United States.

### Data availability.

The raw reads and assembled contigs are available at NCBI BioProject number PRJNA649713.
